# Expression of the 5T4 oncofoetal antigen in renal cell carcinoma: a potential target for T-cell-based immunotherapy

**DOI:** 10.1038/sj.bjc.6602776

**Published:** 2005-09-13

**Authors:** R W Griffiths, D E Gilham, A Dangoor, V Ramani, N W Clarke, P L Stern, R E Hawkins

**Affiliations:** 1 Department of Medical Oncology, Paterson Institute for Cancer Research, Christie Research Centre, Manchester M20 4BX, UK; 2 Department of Immunology, Paterson Institute for Cancer Research, Manchester M20 4BX, UK; 3 Department of Urological Surgery, Christie Hospital NHS Trust, Manchester M20 4BX, UK

**Keywords:** renal cell carcinoma, immunotherapy, 5T4, oncofoetal antigen

## Abstract

The 5T4 oncofoetal antigen is a heavily glycosylated cell surface protein found on human placental trophoblast and on diverse types of human cancer but is not expressed at significant levels on adult human tissues in health. It therefore satisfies the criteria for a tumour-associated antigen and is an ideal target for the immunotherapy of cancer. We report here that 5T4 is strongly expressed on the majority of renal cell carcinomas and therefore this population of patients is suitable for trials of 5T4-targeted therapies. In particular, we have shown that T cells from renal cell carcinoma patients can be genetically modified to kill 5T4 expressing renal cancer cell lines by introduction of a chimeric-signalling protein. This protein consists of a single chain antibody fragment capable of binding antigen directly at the cell surface and then activating the T cell by virtue of a CD3*ζ-*signalling domain. This is a powerful tool that bypasses a number of mechanisms that allow tumours to escape T-cell killing and can be readily scaled up for clinical use.

Renal cell carcinoma (RCC) makes up around 3% of all adult malignant tumours in the United Kingdom and is the commonest neoplasm arising from the kidney (Cancer Registration Statistics, 2001). Approximately a third of patients are cured by surgery while treatment options for metastatic disease are very limited, as the cancer tends to be resistant to both chemotherapy and radiotherapy. Median survival for patients with untreated metastatic disease is around 12 months (Atzpodien *et al*, 2003). The only drugs proven to induce regression of the disease are the immunomodulatory agents interferon-*α* (MRC Renal Cancer Collaborators, 1999) and interleukin-2 (IL-2) (Yang *et al*, 2003). Response rates with these agents are low, in the order of 14–20% and, although a survival advantage has been demonstrated for interferon-*α* over hormonal therapy alone, no clear survival advantage has been demonstrated for IL-2.

Central to the development of better biological therapy for RCC is the need to identify aberrant or overexpressed proteins that may distinguish the tumour from normal tissues. These proteins can then be targeted by a variety of therapeutic strategies such as monoclonal antibodies (Mab), vaccines or adoptive transfer of antigen specific T cells. The goal of these approaches is that the tumour is recognised by the immune system and then eliminated by cytotoxic effector cells. To date only a small number of aberrantly expressed proteins have been associated with RCC. Some of these, such as G250, are cell surface proteins that have been identified by murine Mab (Oosterwijk *et al*, 1986). Clinical trials are already underway utilising a number of immunotherapeutic modalities targeting the G250 protein in RCC (Lamers *et al*, 2002; Bleumer *et al*, 2004).

A number of oncofoetal antigens have now been identified that are expressed in foetal tissues and also by certain malignancies. 5T4 is a 72 kDa glycoprotein identified by a murine monoclonal antibody produced by a hybridoma from splenocytes of mice immunised with syncytiotrophoblast microvillous membrane glycoproteins (Hole and Stern, 1988). It is highly expressed on placental trophoblast and a variety of human cancers (Southall *et al*, 1990). Transduction of the 5T4 cDNA into cell lines enhances cell motility and reduces cell–cell contacts suggesting that it may be mechanistically involved in the malignant phenotype (Carsberg *et al*, 1996). This is further supported by evidence that expression of 5T4 correlates with poorer survival in colorectal carcinoma (Starzynska *et al*, 1994). This protein has only limited expression in normal adult human tissues (Southall *et al*, 1990) making it a useful target for the immunotherapy of cancer.

The most successful targeted approach to immunotherapy undoubtedly has been the use of monoclonal antibody therapy, notably in breast cancer (trastuzumab) (Cobleigh *et al*, 1999) and non-Hodgkin's lymphoma (rituximab) (Czuczman *et al*, 2005). Once bound to their respective target, Mab's induce antitumour activity by one of three principle mechanisms: antibody-dependent cell-mediated cytotoxicity (Clynes *et al*, 2000), interruption of aberrant cell-signalling proteins (Le *et al*, 2000) or delivery of a cytotoxic moiety such as a drug, radioisotope or toxin to the tumour (Wiseman *et al*, 2002). Antibody therapy does, however, have a number of limitations such as poor penetration into tumour, selection of apoptosis-resistant tumour clones and inactivation of Mab by serum proteins (Reilly *et al*, 1995; Jain, 1999). Other strategies for generating an effective immune response against solid tumours such as vaccination and adoptive transfer of antigen-specific T cells are therefore being actively developed.

T cells are one of the key effector cells in a host response to a tumour (Finn and Lotze, 2001), but by the time a cancer has become established, it may have evolved strategies to evade T-cell killing. These include the downregulation of major histocompatibilty complex (MHC) molecules and associated transporter proteins thus preventing antigen presentation of endogenous peptides (Garrido *et al*, 1997) and induction of tolerance in T cells with specificity for tumour-borne epitopes (Mapara and Sykes, 2004). Nevertheless, tumour antigen-specific lymphocytes have been isolated from tumour-infiltrating lymphocytes (TILs) of malignant melanoma (Dudley *et al*, 2002) and these cells were able to reject tumour upon reinfusion following lymphodepleting chemotherapy. It has proved much harder to isolate such cells from RCC tumours (Yannelli *et al*, 1996) and the few studies using adoptive cell transfer are yet to yield any encouraging results (Goedegebuure *et al*, 1995; Figlin *et al*, 1999; Dillman *et al*, 2004).

It may therefore be desirable to evaluate other methods of making T cells that can recognise renal cell cancer. T cells can be genetically modified *in vitro* to possess Mab specificity for a protein epitope by retroviral transduction with a chimeric T-cell receptor (Thistlethwaite *et al*, 2005). This circumvents the natural process of T-cell activation through MHC and means that a large polyclonal pool of T cells can be conferred with specificity for one single antigen. We have previously demonstrated that T cells from patients with colorectal cancer can kill CEA-expressing cell lines when retrovirally transduced with a chimeric T-cell receptor with specificity for CEA protein (Sheen *et al*, 2003). The chimeric T-cell receptor consists of an extracellular recognition domain comprising a single chain antibody fragment; this is then fused to an intracellular CD3*ζ-*signalling domain found in the wild-type TCR. Crosslinking of the chimeric TCR with its respective antigen results in downstream activation of the T cell, which is then able to effect target cell killing either directly by production of cytotoxic moieties such as granzyme, perforin and Fas ligand, or indirectly via production of cytokines important for T-cell killing such as interferon-*γ* (Darcy *et al*, 2000).

Our first aim in this study was to evaluate the expression of the 5T4 oncofoetal antigen by immunohistochemistry in freshly isolated RCC specimens. Secondly, we wished to test its utility as a target for gene modified T cells taken from patients with RCC. Using a chimeric T-cell receptor that binds to the 5T4 protein we have evaluated whether T cells from patients with RCC could be genetically modified to kill allogeneic 5T4-expressing renal cell lines.

## MATERIALS AND METHODS

All tissue and blood samples were collected from three hospitals in the South Manchester region. Approval from the local ethical committee was given prior to sample collection and informed consent was obtained from each patient at least a day before surgery. Additionally, five frozen specimens of normal kidney from donors without malignant disease were obtained from a biomaterials supplier (Medical Solutions, Nottingham, UK). Specimens were obtained post mortem with donor consent and ethical approval.

### Immunohistochemistry

Tumour samples were taken from either primary or metastatic disease in patients with RCC (either suspected or confirmed). Where possible samples of kidney (at a distance of at least 2 cm away from the primary tumour) were also taken and termed ‘normal’ for this study. The histological type and grade of tumour was assessed by routine haematoxylin and eosin staining of paraffin-embedded sections.

Tumour and kidney samples were snap frozen and sectioned using a freezing microtome. A three-stage immunoperoxidase technique using the EnVision kit (DakoCytomation Ltd, Denmark) was used to visualise the 5T4 glycoprotein. Immunohistochemical staining was performed according to the manufacturer's instructions. A murine monoclonal antibody specific for 5T4 was used as a primary antibody (5T4 IgG_1_ 2.2 mg ml^−1^) at a 1 in 1000 dilution. Production and characterisation of this antibody has previously been described (Hole and Stern, 1988). A murine IgG_1_ isotype control was applied at the same concentration to separate sections to act as a negative control.

### Cell culture

The renal carcinoma cell lines were a kind gift from Dr J Yang of the National Institutes of Health, Bethesda, MD, USA. They were cultured as an adherent monolayer in Dulbecco's Modified Eagle Medium with 10% foetal calf serum (Gibco BRL, Paisley, Scotland). T cells were cultured in T-cell media consisting of RPMI-1640 supplemented with 25 mmol l^−1^ HEPES, 50 *μ*mol l^−1^ *β*-mercaptoethanol, 200 mmol l^−1^ L-glutamine, 50 IU ml^−1^ penicillin, 50 *μ*g ml^−1^ streptomycin (all from Sigma, Dorset, UK) and 5% human AB serum (Promocell, Heidelberg, Germany). Cells were kept at 37°C in a humidified atmosphere containing 95% air and 5%CO_2_.

### Isolation and activation of peripheral blood lymphocytes

In all, 50 ml of heparinised blood was taken from patients at the time of surgery. The mononuclear cell fraction was isolated by Ficoll-Hypaque (PAA Laboratories, Pasching, Austria) density centrifugation. The cells were washed twice in RPMI-1640 and resuspended in T-cell media before depletion of monocytes by plastic adherence. After 1 h the flask was removed and nonadherent cells were removed and resuspended in T-cell medium supplemented with 100 IU ml^−1^ of recombinant human IL-2 (Chiron, Amsterdam, The Netherlands) at a concentration of 1 × 10^6^ cells ml^−1^ in T-cell media. The cells were then incubated for 3 days in six-well tissue culture plates precoated with anti-CD3 (Orthoclone, NJ, USA) and anti-CD28 (R&D Systems, MN, USA) Mab. Lymphocytes were then maintained in culture by adding IL-2 every 3–4 days and maintaining cell concentration around 1 × 10^6^ cells.

### Retroviral transduction of peripheral blood lymphocytes

The *Kat* retroviral system (Finer *et al*, 1994) was used to genetically modify the isolated lymphocytes by introducing DNA coding for one of two chimeric T-cell receptors. The retroviral vector rKat.MFE.CD3*ζ*.TDGA.IRES.eGFP (MFE.CD3*ζ*) has been previously described (Gilham *et al*, 2002) and encodes a chimeric T-cell receptor specific for CEA. To create a chimeric T-cell receptor specific for the 5T4 protein, DNA coding for a humanised scFv derived from the mouse anti-human 5T4 monoclonal antibody, together with a human Fc antibody spacer region was exchanged into the above vector. The inclusion of the Fc space was found to be optimal for targeting gastrointestinal cancers expressing 5T4 (Guest *et al*, 2005) and was used in this analysis. This construct 5T4.hFc.CD3*ζ*.TDGA.IRES.eGFP was abbreviated to 5T4.CD3*ζ*. Transduced lymphocytes coexpressed enhanced green fluorescent protein to allow identification of this population by flow cytometry.

The rKat vector was combined with the packaging plasmid pKat and introduced into the 293T packaging cell line by calcium phosphate transfection. Virus was harvested at 48 and 72 h. The first viral harvest was timed to coincide with the end of the 3-day activation period of lymphocytes as described above. Lymphocytes and viral particles were combined in T-cell medium supplemented with 4 *μ*g ml^−1^ polybrene and then centrifuged at 1200 × g for 3 h. This spin-fection was then repeated the following day after the second viral harvest.

### Flow cytometry

Surface markers of *in vitro* cultured cells was analysed on a FACScan (Becton Dickinson, CA, USA) and analysed using CellQuest software. Cells (2 × 10^5^) were resuspended in phosphate-buffered saline supplemented with 2% bovine serum albumin. Phyco-erythrin-conjugated antibodies were used to stain for CD3, CD4 and CD8 (all from Becton Dickinson). 5T4 staining was performed using 1 *μ*g ml^−1^ of murine anti-5T4 followed by phyco-erythrin-conjugated anti-mouse IgG (Sigma, Dorset, UK).

### Chromium release assay

To measure short-term cytotoxicity mediated by T cells a standard 4-h chromium release assay was used. Target cells were incubated for 1 h with 100 *μ*Ci [^51^Cr]sodium chromate, washed three times in serum-free media before resuspending in T-cell media at a concentration of 5 × 10^4^ cells ml^−1^. A total of 5000 target cells per well were then plated out with effector cells in triplicates at the following ratios: 50 : 1, 25 : 1, 12.5 : 1 and 6.25 : 1. Spontaneous release was quantified by adding T-cell medium alone to targets and maximal release by adding 2% Triton to targets. Following 4-h coculture, supernatants were removed and plated on to Luma plates (Packard, Berkshire, UK), released radioactivity was measured using a TopCount plate reader Packard, Berkshire, UK). Percentage-specific lysis of targets was calculated using the following formula: (maximal release−sample release)/(maximal release−spontaneous release) × 100.

### interferon-*γ* release by enzyme-linked immunosorbent assay

Triplicate wells consisting of 1 × 10^4^ RCC cells and 1 × 10^5^ lymphocytes per well in T-cell medium were set up on a 96-well plate. The cocultures were left for 24 h in an incubator at 37°C supplemented with 5% CO_2_. The supernatants were removed and interferon-*γ* concentration determined using matched antibody pairs MAB285 and BAF285 following the protocol defined in the manufacturer's instructions (R&D, Oxfordshire, UK).

## RESULTS

### 5T4 expression on RCC biopsies

**Figure 1:**
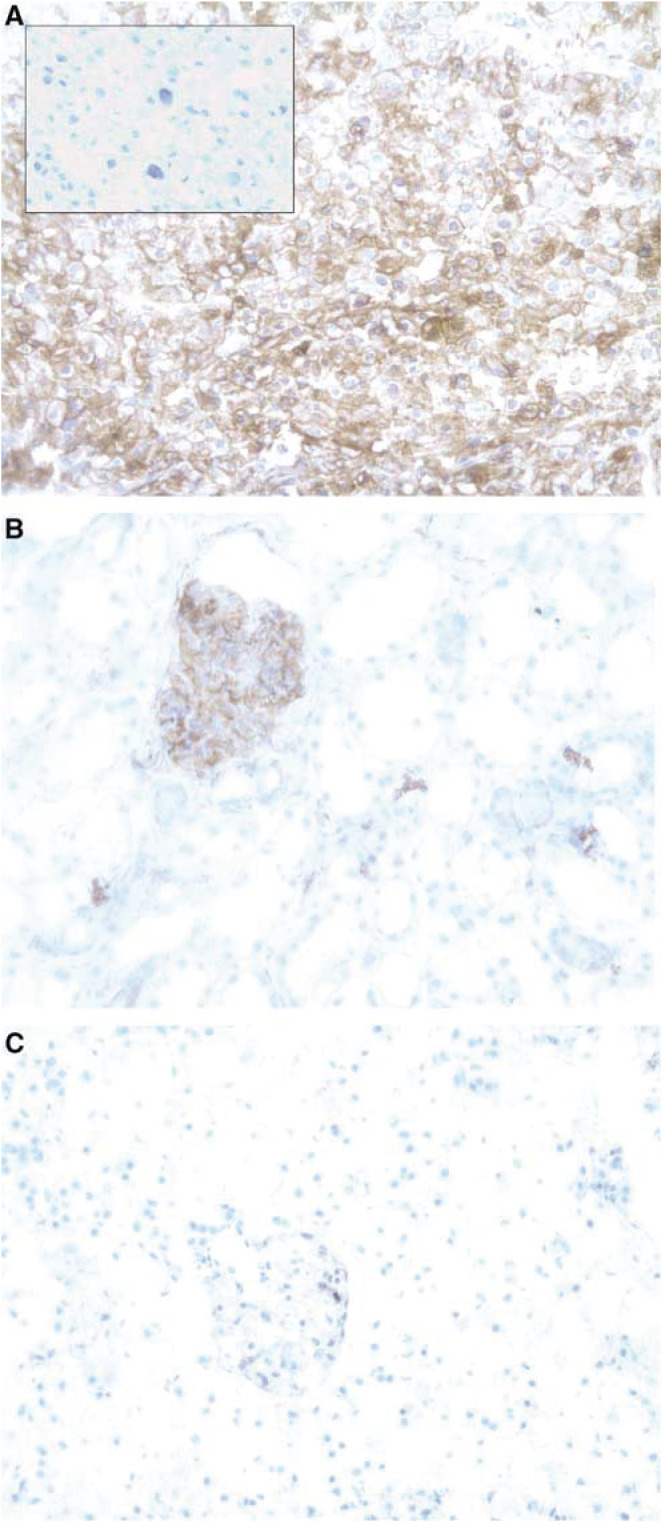
Strong positive 5T4 expression in renal cell carcinoma. Light microscopy (× 20 magnification) of a Fuhrman grade 3 clear cell carcinoma (**A**) following application of murine anti-human 5T4 followed by immunoperoxidase (brown precipitate denoting presence of bound murine antibody) and haematoxylin stains. The inset picture shows the same tissue with a mouse isotype control antibody applied. All malignant tissue examined that stained positive for 5T4 exhibited a membranous staining pattern. A section of kidney taken from the same patient is demonstrated (**B**) showing brown staining within a swollen glomerulus. A sample of tissue from a normal kidney taken from a donor with nonmalignant disease (**C**) was also obtained and this shows much weaker stromal staining within the glomerulus.

Using the EnVision kit, a dark brown precipitate was observed on the specimen if 5T4 was present. Two observers independently examined the specimens under light microscopy and classified them into one of these four groups: strongly positive (++), positive (+), focal staining (+/−) or negative (−) if no staining was observed. The pattern of staining was also characterised as either stromal, tumour membranous or tumour cytoplasmic. This categorisation was used based on the previous patterns of staining noted in colorectal and gastric carcinomas (Starzynska *et al*, 1994). All positive samples however, clearly demonstrated a membranous staining pattern (Figure 1A) and there was no evidence of stromal staining on any of the tumour specimens. Tumour samples were obtained from 20 patients; 18/20 were primary clear cell carcinomas (Table 1), the remaining two being papillary cell carcinomas. 19/20 (95%) samples were at least focally positive for 5T4 and 15/20 (75%) exhibited strong positive staining. From five patients, samples of ‘normal’ kidney at least 2 cm from the resection margin were taken at nephrectomy. Four of the five samples demonstrated strong positive glomerular staining (Figure 1B) while the remaining sample exhibited some focal tubular staining. However, haematoxylin and eosin staining showed an inflammatory infiltrate in all of these specimens and glomerulosclerosis was present in two. The possibility that antigen shedding from the tumour with subsequent deposition in the Bowman's capsule prompted us to evaluate specimens from donors without malignant disease. We examined five samples of normal kidney taken from post mortem specimens. All five demonstrated a weak focal glomerular staining and this was notably less than that seen in the RCC patient ‘normal’ kidney.

### 5T4 expression on RCC lines

**Figure 2:**
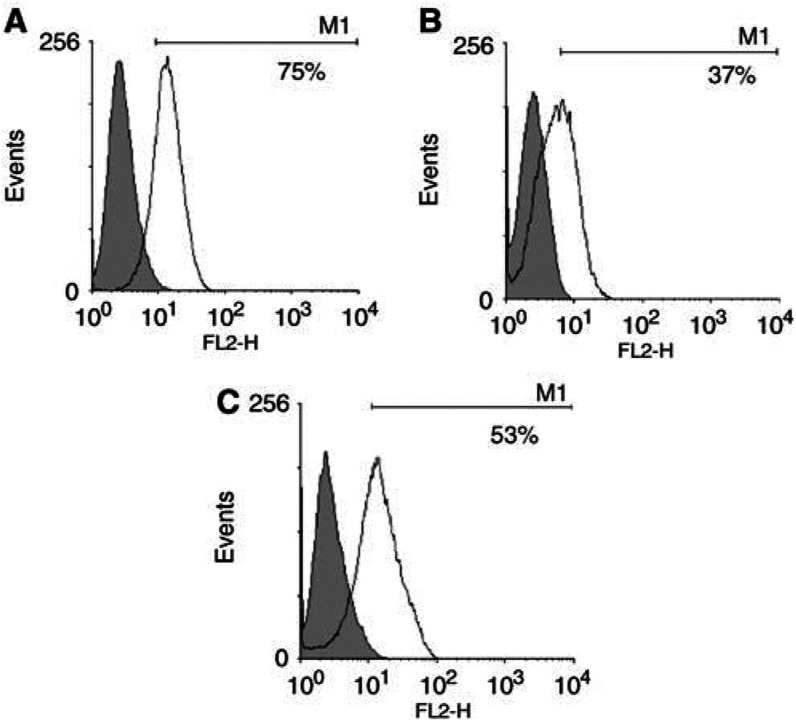
5T4 expression in renal cell carcinoma lines. Renal cell carcinoma cell lines were incubated with murine anti-5T4 antibody or isotype control and then a secondary PE-labelled anti mouse IgG applied. The histograms show the FACS results for (**A**) 2220R, (**B**) 2245R and (**C**) 2246R.

The renal cell lines 2220R, 2245R, 2246R where trypsinised and washed twice in serum-free DMEM. 5T4 expression was evaluated by applying the murine antibody or isotype control and then a secondary phycoerythrin-labelled anti-mouse IgG antibody. All cell lines demonstrated clear expression of 5T4 (Figure 2).

### 5T4.CD3*ζ* transduced lymphocytes demonstrate enhanced killing and interferon-*γ* release when in contact with renal cell lines

Peripheral blood lymphocytes from three patients with RCC were activated on anti-CD3/anti-CD28-coated tissue culture plates as described above. For each patient, a third of the activated T cells underwent a mock transduction with no retrovirus; this population would be used as a negative control to determine background levels of cytotoxicity and cytokine release. A third of the activated lymphocytes were transduced with the MFE.CD3*ζ* construct and the remaining third transduced with the 5T4.CD3*ζ* construct. The RCC cell lines did not express CEA (data not shown) and therefore the purpose of using T cells transduced with the MFE.CD3*ζ* construct was to demonstrate that killing and cytokine release was dependent on the specificity of the 5T4.CD3*ζ* chimeric receptor. We have previously shown the specificity of this receptor for human 5T4 expressed in mouse cell lines (Guest *et al*, 2005). At 3 days prior to performing any of these assays, the IL-2 concentration in the T-cell cultures was reduced to 20 IU ml^−1^ to reduce any background lymphocyte-activated killer (LAK) activity. All assays were performed within 3 weeks of initial transduction.

**Figure 3:**
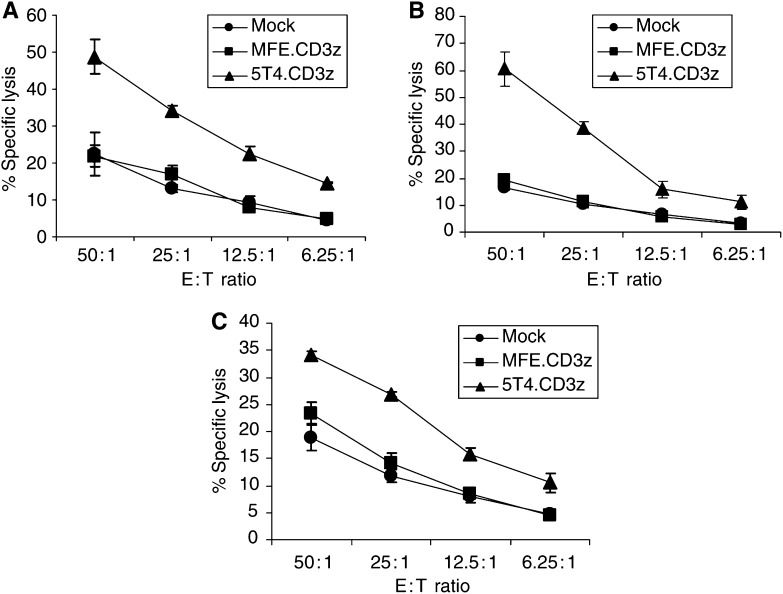
5T4 targeted T cells efficiently kill 5T4 expressing RCC lines. Lymphocytes from patient 4 were activated, expanded and transduced with either the MFE.CD3*ζ* construct, 5T4.CD3*ζ* construct or were subject to a mock transduction. Lymphocyte populations were then cocultured for 4 h with ^51^Cr-labelled RCC cells from cell lines (**A**) 2220R, (**B**) 2245R and (**C**) 2246R. Each point represents a triplicate of wells, each well containing 5000 target tumour cells. The bars refer to the s.d. for each dilution.

**Figure 4:**
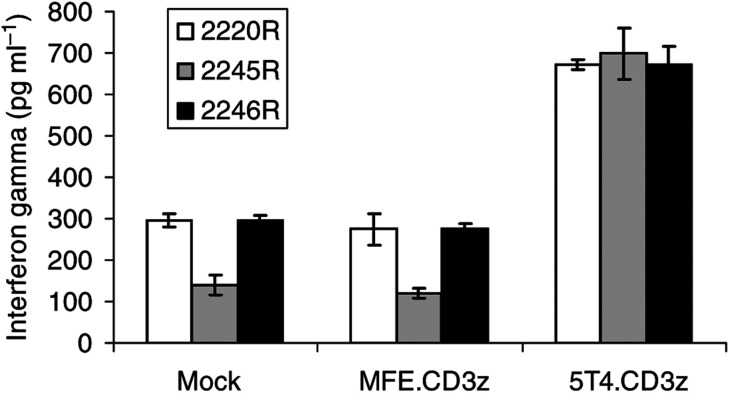
Interferon-*γ* release. Representative results again from patient 4. In all, 1 × 10^5^ lymphocytes were cocultured for 24 h with 1 × 10^4^ RCC cells from the three cell lines: 2220R, 2245R and 2246R. After 24 h the supernatants were removed and assayed for interferon-*γ* by ELISA. Error bars refer to the s.d. for each dilution.

Prior to each assay being performed, the lymphocyte populations were analysed by flow cytometry. All lymphocyte populations were >92% CD3+ve indicating they were predominantly T cells (Table 2). In patients 4, 11 and 14 the transduction levels of MFE.CD3*ζ* were 43, 38 and 24%, respectively. However, the values for 5T4.CD3*ζ* were significantly lower for each patient at 22, 15 and 13%, respectively (Table 2). We have routinely found that efficiency of expression of the 5T4.CD3*ζ* construct is approximately half the level of the MFE.CD3*ζ* construct although the reason for this is not clear. A 4 h chromium release assay was performed on each of the three RCC lines and in each case, we observed significantly enhanced killing of the renal cell lines by 5T4.CD3*ζ*-transduced lymphocytes when compared to either the MFE.CD3*ζ* transduced or the mock-transduced lymphocytes (Figure 3). To determine functionality of the modified T cells in mounting an effective cytokine response, the production of interferon-*γ* from T cells was assayed when cocultured with target cells at a ratio of 10 : 1. Figure 4 demonstrates that the 5T4.CD3*ζ*-transduced T cells were able to generate significantly higher amounts of interferon-*γ* on contact with 5T4 expressing cell lines compared to either mock transduced or MFE.CD3*ζ*-transduced T cells. Importantly, the levels of cytotoxicity and interferon-*γ* release in the mock and MFE.CD3*ζ*-transduced cells were similar indicating that this effect had not occurred as a result of retroviral transduction.

## DISCUSSION

We have demonstrated in twenty RCC specimens that the 5T4 oncofoetal antigen is expressed to high levels in almost all cases of RCC. Importantly, the strong positive membrane expression on most of the tumour specimens makes RCC a good candidate for 5T4-targeted therapies. Previous reports of 5T4 have documented minimal expression on normal tissue (Southall *et al*, 1990) and we wished to confirm this was the case with respect to the kidney. We were able to analyse normal kidney taken greater than 2 cm from the tumour margin in five patients, however, the surrounding kidney in RCC frequently shows inflammatory change and our samples were no exception. We observed the presence of 5T4 within the Bowman's capsule in all five of our ‘normal’ kidney specimens. In case this result was due to antigen shedding and glomerular deposition, we also examined five post mortem specimens from donors who had otherwise healthy kidneys and no known malignant disease. They also showed some evidence of focal glomerular 5T4 expression although to a much lesser extent. It would appear therefore; that there is low-level expression of 5T4 on renal glomeruli and this confirms a previously published result (Connolly *et al*, 2003; Valle *et al*, 2003). A number of immunological therapies targeting 5T4 are currently undergoing assessment in phase I and II clinical trials and it should be noted that in no patients to date has any renal toxicity been reported. 5T4 has been targeted by a vaccine-based approach: A gene-modified vaccine has been developed by inserting the gene for 5T4 into an attenuated vaccinia virus (Trovax™). In preclinical murine models a similar construct demonstrated protection against tumour establishment and activity against pre-existing tumour (Mulryan *et al*, 2002). TroVax has been used clinically in phase I and II trials for patients with metastatic colorectal cancer and can induce antibody and T-cell responses to 5T4 with no toxicity (Valle *et al*, 2003). This finding is of relevance to RCC as this is one of few diseases were positive clinical results have been observed using a vaccine-based approach. A recent German trial has demonstrated improved disease free-survival with an adjuvant tumour cell vaccine in high-risk RCC patients (Jocham *et al*, 2004).

Another approach utilising 5T4 involves generation of a monoclonal antibody immune conjugate. Here, a monoclonal antibody against 5T4 is linked to a superantigen such as *Staphylococcal enterotoxin* B that directly binds the T-cell receptor in a construct engineered to reduce its MHC class II binding. Thus, the fusion protein acquires the ability to activate T cells when binding to the 5T4 target on the tumour cell. Accumulation of this immune conjugate at the tumour site should then result in local T-cell recruitment and activation (Connolly *et al*, 2003).

It has been recognised that the 5T4 antigen is present on a wide range of tumour types and that its expression can be associated with a poorer prognosis. In our study, we have insufficient numbers to correlate the degree of expression to prognosis or histological grade. Previous studies have shown that the human 5T4 protein when transduced into cell lines reduces cell–cell contacts and enhances cell motility, suggesting it may play a role in supporting tumour invasion and metastasis (Carsberg *et al*, 1995, 1996). The remarkable finding in this study is the consistency of high expression levels of 5T4 in RCC and it is therefore tempting to speculate whether this may be linked to other proteins expressed to high levels in RCC. A frequent finding in the majority of conventional clear cell carcinomas is a mutation in the von Hippel-Lindau (VHL) suppressor gene (Ma *et al*, 2001), the protein product of this gene is required for degradation of hypoxia-inducible factor 1*α* (HIF-1*α*). Tumour hypoxia and VHL gene mutations in RCC lead to accumulation of HIF-1*α* (Wiesener *et al*, 2001), this protein then leads to transcription of a number of genes which predominantly affect angiogenesis and assist in tumour expansion. The human 5T4 promoter sequence has been examined and does not appear to contain any sequences that are now known to be HIF-1*α* responsive (Myers *et al*, 1994), but this is an area which needs further exploration, particularly because HIF-1*α* is instrumental in mediating early trophoblast differentiation. Our main interest in this protein, however, is that it represents a useful tumour antigen in that it is expressed strongly at the cell surface in RCC tumours and minimally in normal tissues. We did observe focal glomerular staining within surrounding kidney in one of the five patients from whom normal tissue could be obtained. We therefore propose that because of the consistent expression of this protein in RCC and the sensitivity of this tumour to immunological therapies, future trials of 5T4-targeted agents could particularly focus on this population of cancer patients.

The use of adoptively transferred T cells as a therapy for solid tumours is still in its infancy, with one of the main barriers to the technique being the ability to generate large numbers of tumour antigen-specific lymphocytes. Large numbers of tumour reactive lymphocytes isolated from naturally occurring TIL populations have been used for the treatment of melanoma and clinical responses have been observed in patients who had already failed multiple lines of conventional therapy (Dudley *et al*, 2002). However, in one case report, resistance to treatment occurred as a result of the therapy selecting out tumour cells which lack MHC I expression (Khong *et al*, 2004). Isolation of tumour-specific clones of T cells is possible with melanoma because of common presence of skin metastases and identification of common T-cell antigens; obtaining and expanding such cells from other solid tumours has been more difficult. The ability to engineer T cells from the peripheral blood and confer on them antibody-type specificity without the need for MHC presentation of antigen by tumour represents an important step forward in bypassing some of the barriers to effective T-cell therapy. This modality is also likely to have a number of advantages over conventional monoclonal antibody therapy in that T cells have discrete homing and tissue penetration capabilities together with the ability to effect immediate target cell death on activation via their T-cell receptor. As a result of the potency of the T-cell response, one major concern is that of reactivity against normal host cells that also express the protein that is being targeted. Evidence to date suggests that 5T4 is expressed at low levels in normal subjects but a carefully planned phase I study is now appropriate.

Adoptive T-cell therapy is of particular relevance to RCC given the sensitivity of this tumour to immunomodulatory drugs. The ability to generate 5T4-specific T cells *ex vivo* represents an important advance in bringing this modality of treatment into the clinic. Before this technique is scaled up for clinical use, there remain issues yet to be resolved. Firstly, the 5T4.CD3*ζ* construct has a comparatively poor transduction efficiency using the *Kat* retrovirus as described, and this needs to be optimised. Secondly, the optimal method of expanding T cells for transduction is still being debated. We routinely use IL-2 to expand T cells, but evidence is emerging that this may also assist in the expansion of regulatory T cells, this subpopulation may be impeding some of the observed cytotoxicity that we are engineering in to these cells (Nelson, 2004). There may be advantages in using different cytokines such as IL-7 and IL-15 to grow cytotoxic T cells (Antony and Restifo, 2005), or alternatively magnetically depleting regulatory cells from the precursor population. Another development in the field of T-cell expansion is the use of T-cell expansion beads coated with antibodies to CD2, CD3 and CD28 (Dudley, 2003). These appear to provide more reliable and rapid expansion of T cells and may therefore improve transduction efficiency and also generate higher numbers of cells that are needed for adoptive transfer.

In conclusion, we have shown that polyclonal lymphocytes taken from the peripheral blood can be directed against the 5T4 protein strongly expressed on the vast majority of RCCs. This method could lead to an effective approach for the cellular therapy of this notoriously difficult disease.

## Figures and Tables

**Table 1:** Patient characteristics and 5T4 expression

**Sex**	**Age**	**Nature of specimen**	**Histology**	**Fuhrman grade**	**TNM stage**	**Pattern of 5T4 staining**
F	67	Primary renal	Papillary	2	T2N0M0	++
M	67	Primary renal	Papillary	2	T1bN0M0	++
M	70	Primary renal	Clear cell	2	T3aN0M0	+/−
F	53	Primary renal	Clear cell	3	T3aN0M0	−
F	67	Primary renal	Clear cell	3	T3aN0M0	++
F	48	Primary renal	Clear cell	2	T1aN0M0	+
F	68	Primary renal	Clear cell	2	T1bN0M0	++
M	57	Primary renal	Clear cell	4	T4N2M1	++
M	73	Primary renal	Clear cell	2	T1bN0M0	++
M	50	Primary renal	Clear cell	1	T1bN0M0	++
M	49	Primary renal	Clear cell	4	T3bN0M0	+
M	75	Primary renal	Clear cell	3	T3bN0M0	+/−
M	64	Primary renal	Clear cell	2	T3bN0M0	++
F	46	Primary renal	Clear cell	2	T3aN0M0	++
F	65	Primary renal	Clear cell	3	T3bN0M0	++
M	64	Primary renal	Clear cell	2	T3bN0M0	++
F	43	Metastasis	Clear cell	NA	TXNXM1	++
M	66	Metastasis	Clear cell	NA	TXNXM1	++
M	49	Primary renal	Clear cell	3	T1aN0M0	++
F	51	Primary renal	Clear cell	2	T3aN0M0	++

NA=not applicable.

**Table 2:** Phenotype of transduced lymphocytes

	**CD3 (%)**	**CD4 (%)**	**CD8 (%)**	**EGFP (%)**
*Patient 4*				
Mock	94	50	36	NA
MFE.CD3*ζ*	95	50	35	43
5T4.CD3*ζ*	92	40	36	22
				
*Patient 11*				
Mock	98	35	60	NA
MFE.CD3*ζ*	99	31	57	38
5T4.CD3*ζ*	99	35	58	15
				
*Patient 14*				
Mock	94	15	68	NA
MFE.CD3*ζ*	96	18	64	24
5T4.CD3*ζ*	95	22	65	13

NA=not applicable.
